# A vicious cycle: employment challenges associated with diabetes foot ulcers in an economically marginalized Southwest US sample

**DOI:** 10.3389/fcdhc.2023.1027578

**Published:** 2023-04-14

**Authors:** Kelly N. B. Palmer, Rebecca M. Crocker, David G. Marrero, Tze-Woei Tan

**Affiliations:** ^1^Department of Health Promotion Sciences, Mel and Enid Zuckerman College of Public Health, University of Arizona, Tucson, AZ, United States; ^2^Center for Health Disparities Research, University of Arizona Health Sciences, Tucson, AZ, United States; ^3^Division of Vascular Surgery and Endovascular Therapy, Keck School of Medicine, University of Southern California, Los Angeles, CA, United States; ^4^Department of Surgery, University of Arizona College of Medicine, Tucson, AZ, United States

**Keywords:** diabetic food ulcers, diabetes management, employment challenges, health equity, patient – centered care, multisectoral approach, patient perspective, social determinants of health

## Abstract

**Aim:**

To describe patients’ reported employment challenges associated with diabetic foot ulcers (DFUs).

**Methods:**

Fifteen patients from under-resourced communities in Southern Arizona, with a history of DFUs and/or amputations, were recruited from a tertiary referral center from June 2020 to February 2021. Participants consented to an audio-recorded semi-structured phone interview. Interviews were transcribed and thematically analyzed using the Dedoose data analysis platform.

**Results:**

Participants shared a common theme around the cyclic challenges of DFU prevention/management and employment. Those employed in manual labor-intensive jobs or jobs requiring them to be on their feet for long durations of time believed working conditions contributed to the development of their DFUs. Patients reported work incapacity due to declines in mobility and the need to offload for DFU management. Many expressed frustration and emotional distress related to these challenges noting that DFUs resulted in lower remuneration as medical expenses increased. Consequently, loss of income and/or medical insurance often hindered participants’ ability to manage DFUs and subsequent complications.

**Conclusion:**

These data illuminate the vicious cycle of DFU and employment challenges that must be addressed through patient-centered prevention strategies. Healthcare providers should consider a person’s contextual factors such as employment type to tailor treatment approaches. Employers should establish inclusive policies that support patients with DFUs returning to work through flexible working hours and adapted work tasks as needed. Policymakers can also mitigate employment challenges by implementing social programs that provide resources for employees who are unable to return to work in their former capacity.

## 1 Introduction

Over 30 million adults in the United States are living with diabetes, a physically, emotionally, and economically burdensome disease (1). It is a complex disease requiring sophisticated clinical and self-management. Without proper management and support, people with diabetes are at risk for acute and chronic complications that lead to increased morbidity and mortality. Among these complications are diabetic foot ulcers (DFUs), a leading cause of hospital admissions and amputations for patients with diabetes (2–4). Even though 75% of DFUs are potentially preventable, in the United States, a quarter of patients with diabetes will endure a foot ulceration in their lifetime (5, 6). Having a DFU more than doubles a person’s five-year mortality rate (3, 7). Patients with DFUs have reported various barriers to DFU prevention and management such as insufficient insurance coverage, delays in receiving clinical care, diabetic footwear, and wound care supplies, and financial strain (8).

There are multiple risk factors that can contribute to the development of DFUs in persons with diabetes, including prolonged hyperglycemia, increased BMI, pressure from footwear, low SES, and smoking (9–11). In this study, we sought to ascertain the factors that groups that have been economically marginalized and people from racial-ethnic minority groups based in the southwestern U.S. perceived as contributing to the onset of and complications resulting from DFUs. We have previously reported results related to the pernicious roles of substandard healthcare access and services (8), lack of awareness surrounding DFUs and care regimens, and poor sensory perception related to neuropathy (12) in the onset and poor outcomes from DFUs. In addition, we have highlighted the multiple and burdensome impacts of DFU as related by study participants, including emotional suffering, complex care regimens, declines in ambulatory function, and financial strain (13).

Economic instability has been cited elsewhere as both a contributing factor of and a resulting stress from DFUs (8, 13). DFUs can hinder one’s ability to work which in turn threatens a patient’s access to medical care and resources needed to care for DFUs. In contrast, employment status or type may increase a person’s risk for developing DFU. Individuals who work long hours or perform physically demanding jobs may lack the autonomy or flexibility required for DFU prevention and management. On the whole, patient groups that have been economically marginalized endure an inequitable burden of DFUs and amputations and death related to DFUs (14).

Historically, research has focused on the economic impact of diabetes to society through loss of productivity and reduced workforce. It is well documented that patients with diabetes suffer from absenteeism, under employment, and unemployment at higher rates than those without diabetes (15–17). Further, complications such as diabetic foot ulcers have been shown to be major causes of disability and premature retirement (18). However, we know less about how patients perceive employment challenges and how those challenges impact patients’ ability to manage their diabetes and prevent and manage DFUs. To our knowledge, this is the first study to qualitatively explore patients’ perceptions of DFU prevention and management in the context of employment. This article describes the ways in which employment and financial challenges impacted both initial onset of DFUs and subsequent management as well as the impact of DFUs on employment performance and status.

## 2 Methods

### 2.1 Study design

This qualitative descriptive study aimed to understand the contextual factors impacting patients’ ability to manage their diabetic foot ulcers as well as examine the effects of diabetic foot ulcers on their quality of life. Using a qualitative study design allowed us to capture detailed personal accounts and insights from patients that otherwise may not have been obtained through quantitative measures.

### 2.2 Participants

Fifteen patients with a history of DFUs and/or amputations were recruited from the Southern Arizona Limb Salvage Alliance (SALSA), a tertiary referral center in Southern Arizona from June 2020 to February 2021. There are more than 5,000 patient visits at SALSA annually for diabetes-related foot problems. Of the patient population at SALSA, 40% identify as Native American or Hispanic. In addition, SALSA primarily cares for patients from under-resourced communities and groups struggling against economic marginalization. Patients who had a history of new DFU(s) (less than 6 months) and/or amputation(s) were identified. During their next scheduled clinic appointment or by phone call, they were approached for participation by a research coordinator. We used criterion sampling to purposely recruit diverse participants based on race, ethnicity, gender, history of ulcers, and amputations. Patients who were interested provided written informed consent and were contacted to schedule an interview.

### 2.3 Interview guide development

The research team collaboratively developed an interview guide in an iterative process that was informed by team reflections following the first three interviews. Participants were asked about what they believed caused their DFU, sources of stress related to their DFU, challenges with managing treatment for their DFU, and the impact of having a DFU on their daily life and activities. The study protocol and all relevant materials were submitted to and approved by the University of Arizona Institutional Review Board (1906749805).

### 2.4 Data collection

Originally, focus group discussions were the planned methodology, but due to COVID-19 restrictions for in-person data collection, the team switched to conducting semi-structured interviews *via* telephone and Zoom. Through these interviews, we were able to further elucidate the perceptions, experiences, and opinions of patients as to why they had developed DFUs. Our intention was to use these data to help inform clinical practice and improve patient outcomes. Participants consented to an audio-recorded interview *via* the Tape A Call (www.tapeacall.com) mobile application or the University of Arizona Health Sciences Zoom platform. Participants who were interviewed *via* Zoom had the option to use the video (with the camera on or off) or call-in feature. Interviews were conducted in the participant’s preferred language of English or Spanish by three non-clinical members of the research team (R.M.C., K.N.B.P., and D.G.M.) with experience conducting qualitative interviews. Interviews were, on average, 50 minutes in duration. To reduce barriers to participation, participants were scheduled at a day and time that was most convenient for them (e.g., mornings, evenings, weekends). The study team met regularly to share notes and reflections from the interviews.

### 2.5 Analysis

Audio files of the interviews were professionally transcribed in the language spoken. Transcripts from interviews conducted in Spanish were translated to English. Quality assurance checks were conducted by listening to the original audio file and checking for accuracy of the transcript noting any discrepancies. Transcripts were then uploaded to the Dedoose (www.dedoose.com) data analysis application and independently coded by three members of the research team (R.M.C., K.N.B.P., and T-W.T.). During regular team meetings, codes were revised, and discrepancies were resolved through discussion and consensus. Themes were identified and finalized when agreement over thematic saturation was reached by the coders and with consultation with the clinical member of the research team.

## 3 Results

**Table 1** summarizes participant demographics. Fifteen participants consented and completed an interview. The mean age of participants was 54.2 years. We intentionally enrolled equal numbers of participants identifying as Native American, Hispanic, and white. The sample included five women and 10 men. While all the participants had a history of at least one DFU, most also had a history of foot infection (n=12) and minor or major amputation (n=9). The majority of participants (n=11) were recipients of government-subsidized healthcare through Medicaid or Indian Health Service and were either unemployed or retired (n=12). A pervasive theme among participants centered on the cyclic challenges of DFU prevention/management and employment.

**Table 1:** Participant demographics (N=15).

Characteristics	Patients (N=15) (%)
**Age, year**	54.2
Gender	
Male Female	10 (66.7%) 5 (33.3%)
Race/ethnicity	
White Native American Hispanic	5 (33.3%) 5 (33.3%) 5 (33.3%)
Primary Insurance	
Commercial Medicare Medicaid or Indian Health	1 (6.7%) 3 (20.0%) 11 (73.3%)
Employment Status	
Employed Unemployed Retired	3 (20.0%) 7 (46.7%) 5 (33.3%)
History of Diabetes Related Foot Problems	
History of Diabetic Foot Ulceration	15 (100.0%)
History of Diabetic Foot Infection	12 (80.0%)
History of Peripheral Artery Disease Open surgery or endovascular procedure	7 (46.7%) 4 (26.7%)
History of Minor Amputation	8 (53.3%)
History of Major Amputation	1 (6.7%)

### 3.1 Work as a causal factor of DFU onset

Participants who were currently or previously employed talked about the type of work they did and its contribution as a cause of the DFU. Many of them are/were restaurant servers, construction workers, farm workers, or worked other high-labor jobs that required them to be on their feet for extended periods of time, put repeated and sustained pressure on their feet, or in environments where their feet were exposed to high amounts of moisture due to sweating from the Arizona heat. One participant describes their belief that working long hours resulted in a DFU despite having shoe ware designed to prevent DFUs.

*“I think maybe the shoes I was wearing, I do have diabetic shoes, but I was … I buy extra wide tennis shoes and extra wide work shoes for that reason. And so maybe they weren’t wide enough. I’ve been wearing them for about a year and a half. But I just think the extra prolong time on my feet that particular week is what probably caused it. Like I said, in my mind, I’m thinking my foot used to that kind of work that many hours on my feet, but that one, I probably extended it three or four hours more each night for about seven days. And so, I think didn’t get enough opportunity to recover and then it formed an ulcer.”*

### 3.2 DFUs as a cause of work absenteeism or incapacity

Participants also discussed how the challenges of managing their DFU led to work absenteeism and reduced their ability to resume the full range of regular work activities. For many, the lengthy healing process, the need to offload, and declines in ambulatory function significantly impacted their ability to work. One participant was concerned with their job security due to the amount of time they would be off work and the uncertainty of returning to their place of employment.

“*I said, my job is still working, waiting this year. You know, how long this is going to be? I have not discussed anything that we talked about the fact that I may not be returning. Or if I do, it’s going to be a different capacity, I suppose. But that’s, in my mind. I think that’s not something to discuss, because I really don’t know. The only thing I know for sure, as my surgeon said, I would be off my feet for two months. Right out of the hospital. So that is what I’ve told my work.”*

Another participant echoed the sentiment regarding the extensive timeframe for their DFU to heal.

*“Like I said, work, activities, down time … yeah, it was several months, I think it went for like 7-8 months … I was not too thrilled about that.”*

For some participants, offloading not only affected their ability to work, but also resulted in emotional stress. Patients expressed frustration with not being able to work.

*“I’m not working right now, but I wish [I] was.”*

One participant talked about limited mobility and not being able to engage in physical activity which is recommended for diabetes self-management due to having to offload for their DFU.

*“I get ulcers every now and then because it’s hard to offload the foot. And because I have an open sore, I can’t go swimming, I can’t go for walks when I want to, you know? I try not to dwell on it because it is what it is.”*

Participants who were employed were met with deciding in what capacity they would return to the workforce.

This participant told us their doctor suggested they would not be able to return to their previous employment that required them to be on their feet a lot.

*“When it comes to work … I think it’s best that you start thinking about changing what you do … I was like, why? am I going to be in a wheelchair? Am I going to be on with a cane? Am I not able to stand [up]? I will not be able to spend the amount of hours I’ve spent in the past on my feet.”*

Another participant contemplated making a career change working fewer hours in a day/week that would be advantageous for DFU prevention/management and overall quality of life but also considered the economic consequences to their family of such a decision.

*“I’ve been thinking of getting out of the business, prior to this going on. I’ve talked about it for years (laugh). I’m at the age now that, you know, I have a son [and] a wife, and I never see him based on my hours. And I’ve talked and talked about trying to find a different kind of job that would allow that to happen. I guess, for lack of a better word, a more normal hour type job eight hours a day, you know, 40 hours a week, which I’ve never worked in my life, it’s always been 65 hours or up. And so, when this happened when I went into the hospital prior to knowledge of what, where I’d be right now, I’d mentioned to my wife that maybe this is this is a time to make that adjustment to find a different job. You know, the pay I make is pretty substantial. I make great money where I’m at. And we’ve always known that if I make a job change, it’s not going to be the same. I mean, maybe there’s a chance that I can find something [else], but most likely [not, because of] Corona [COVID-19].*

A couple of participants discussed absenteeism as entrepreneurs. These participants were either the primary or sole operator of their business. There were no paid leave benefits to cover time off resulting in loss wages. One participant recalled feeling conflicted over deciding to stay home and offload to allow the DFU to heal or to go to work and maintain their income.

*“…had a big impact on my work. I’m self-employed so if I’m not working then I don’t get paid. I don’t have [a regular salary], you know what I mean. So that was pretty hard to stay home”*

This participant, who underwent an emergency minor amputation, expressed how concerned they were for their small business’ survival after being unexpectantly shut down for months.

*“I came to work, I put a note on my door that said that I would be opening at 1 o’clock in the afternoon, because I figured that would give me plenty of time [to get back]. And four months later, I came and unlocked the door…”*

### 3.3 The financial impact of DFU-related job insecurity

The final arc of the vicious cycle between DFUs and employment challenges was how under and un-employment tied to DFU healing and immobility in turn further constricted participants’ ability to heal from DFU due to financial barriers. Participants relied on employment to afford diabetes-related expenses such as proper footwear and access to medical care. The work incapacity they faced due to declines in mobility and the need to offload for DFU management resulted in lower remuneration at a time when they incurred higher medical expenses.

*I became medically retired and I’ve lost more than half of my income…. And then I go to the doctor, quite often, a couple … sometimes even three times a week … You know, my wife has to take work off and it’s a pain….*

*I am currently [unemployed] … social security, it’s not enough to cover the copay^1^…They have been charging me, but I don’t have the money to pay it.*

Consequently, loss of income and/or insurance further hindered their ability to manage their DFUs and subsequent complications. Participants expressed frustration and emotional distress with work absenteeism for themselves and caregivers to attend doctor’s appointments sometimes forgoing a visit to the doctor due to work obligations.

## 4 Discussion

Diabetic foot ulcers pose a major threat to the employment viability of patients with diabetes. Conversely, employment contexts such as length of shift, type of work performed, work conditions and environment can contribute to or exacerbate DFUs. Patients with DFUs are met with the difficulties of maintaining employment while trying to prevent and/or manage DFUs. These data illuminate the complexities and vicious cycle of employment challenges associated with the diabetes foot that must be addressed through patient-centered prevention strategies and coordinated care.

Offloading (relieving pressure) is the basic principle of DFU management (19). The preferred offloading treatment for neuropathic DFU is to use total contact cast or offloading device. In individuals with peripheral neuropathy (without active DFU), excessive plantar pressure, especially among those without appropriate diabetic footwear, has been associated with the onset of foot ulceration (20, 21). Given the labored nature of employment many participants engaged in, it is not surprising that participants emphasized working conditions as causal factors of DFUs and inhibited DFU healing due to the inability to offload the DFU. Some participants worked outdoors or were required to wear protective footwear that caused excessive sweating putting them at risk for blisters and calluses. Other jobs required weight-bearing activities that also put undo strain on participants’ feet resulting in DFUs and preventing them from returning to such job duties.

Previous studies have reported that people with diabetes are more likely to be unemployed or underemployed, have higher rates of absenteeism and early retirement (18, 22, 23). Our study sample mirrored these findings as most were unemployed or retired. Participants in our sample that were employed or previously employed had experienced high rates of work-loss days per year. Many of the patients in our study were hourly employees or shift workers who had little to no paid leave benefits to cover time away from work for doctor’s visits or to allow for DFUs to heal. For those with DFUs and other diabetes comorbidities, not having covered time to attend the high volume of medical appointments oftentimes resulted in delayed care seeking, diagnosis, and/or compromise to treatment protocols.

Participants struggled financially when employment was threatened due to DFUs. Most participants were beneficiaries of Medicaid or Indian Health Services, but noted that their diabetes-related care was a financial stress for them (13). Being unemployed or underemployed was a barrier to care for these participants. Many could not afford the wound dressings, medications, and supplies necessary to properly care for their DFUs. Further, some patients had difficulty with expenses for medical visits either because they lived far distances from where they received care, or they had multiple co-pays for each of the many specialists treating their DFUs.

To better support patients prevent and/or manage DFUs, healthcare providers should consider a patient’s contextual factors such as employment type to create a tailored approach to education that addresses unique issues including those detailed here. In addition, patients would benefit from efforts to facilitate access to proper affordable footwear and flexible scheduling options for clinical appointments. Employers should establish inclusive policies that support people with DFUs returning to work through flexible work hours and adapted tasks as needed. Acknowledging the needs and limitations of employers, policymakers can also mitigate employment challenges by implementing social programs that provide transportation access, supplemental income to attend doctor’s appointments for those without paid leave and through temporary employment placement and/or training opportunities for employees that are unable to return to work in their former capacity.

There are limitations to this study for consideration. The small sample size and overrepresentation of male patients limits generalizability. However, the sample is representative of Native American and Hispanic patients, two economically marginalized groups that suffer a disproportionate burden of diabetes-related limb amputation (24). Further, the patients in this study were mostly unemployed and/or employed in high-labor jobs that compounds the existing disparities for patients from these communities. The high prevalence of manual labor with lower economic security further emphasizes the importance of investigating context when deciding on a course of treatment and patient education.

Results from this study provide contextual insight into the challenges patients face with employment and DFU prevention and management. The qualitative data presented here supports previously published quantitative economic and quality of life literature which has demonstrated that patients who experience diabetes-related complications such as DFUs need support to remain engaged in the workforce. We provide recommendations for multisectoral, patient/person-centered approaches to mitigate DFU prevention and management and enhance employment sustainability.

## Data availability statement

The datasets presented in this article are not readily available because of patient confidentiality and participant privacy. Requests to access the datasets should be directed to the corresponding author.

## Ethics statement

This study involving human participants was reviewed and approved by the University of Arizona Institutional Review Board. The patients/participants provided their written informed consent to participate in this study.

## Author contributions

KP: Directed methodology, conducted interviews, coded and analyzed data, wrote manuscript. RC: Directed methodology, translated interview guide and transcripts, conducted interviews and coded and analyzed data. DM: Directed conceptualization of study and methodology, and conducted interviews. T-WT: Acquired funding, directed conceptualization of study, recruited patients, and analyzed data. All authors contributed to the article and approved the submitted version.

## Footnotes

1 a contribution made by an insured person toward the cost of medical treatment or other services.
